# FTO in health and disease

**DOI:** 10.3389/fcell.2024.1500394

**Published:** 2024-12-18

**Authors:** Daniel Benak, Anezka Sevcikova, Kristyna Holzerova, Marketa Hlavackova

**Affiliations:** Laboratory of Developmental Cardiology, Institute of Physiology of the Czech Academy of Sciences, Prague, Czechia

**Keywords:** FTO, m^6^A, m^6^Am, obesity, diabetes, cardiovascular disease, cancer

## Abstract

Fat mass and obesity-associated (FTO) protein, a key enzyme integral to the dynamic regulation of epitranscriptomic modifications in RNAs, significantly influences crucial RNA lifecycle processes, including splicing, export, decay, and translation. The role of FTO in altering the epitranscriptome manifests across a spectrum of physiological and pathological conditions. This review aims to consolidate current understanding regarding the implications of FTO in health and disease, with a special emphasis on its involvement in obesity and non-communicable diseases associated with obesity, such as diabetes, cardiovascular disease, and cancer. It also summarizes the established molecules with FTO-inhibiting activity. Given the extensive impact of FTO on both physiology and pathophysiology, this overview provides illustrative insights into its roles, rather than an exhaustive account. A proper understanding of FTO function in human diseases could lead to new treatment approaches, potentially unlocking novel avenues for addressing both metabolic disorders and malignancies. The evolving insights into FTO’s regulatory mechanisms hold great promise for future advancements in disease treatment and prevention.

## 1 Discovering FTO and its function

In 1999, *Fto* was identified as one of the several genes deleted in mice with the *Ft* (Fused toes) mutation. It was named Fatso due to its large size, which is where the abbreviation *Fto* comes from (Peters et al., 1999). However, its role and function were unknown for a long time. In 2007, human genome-wide association studies (GWAS) revealed that single nucleotide polymorphisms (SNPs) in the human *FTO* gene were associated with increased body mass index (BMI) and obesity, which is where the gene and its product derive their full name now: fat mass and obesity-associated (Hinney et al., 2007; Frayling et al., 2007; Scuteri et al., 2007).

The initial mechanistic insights into FTO’s function emerged already in 2007, demonstrating that FTO could catalyze the 2-oxoglutarate-dependent oxidative demethylation of 3-methylthymine in single-stranded DNA and 3-methyluracil in single-stranded RNA *in vitro* (Gerken et al., 2007; Jia et al., 2008). However, the main biological substrate of FTO was unknown until 2011, when it was reported that FTO had efficient oxidative demethylation activity towards N^6^-methyladenosine (m^6^A), an abundant mRNA modification known from the 1970s (Jia et al., 2011; Desrosiers et al., 1974). This breakthrough discovery revealed the dynamic nature of mRNA modifications and renewed the interest of scientists in this field (Dieterich et al., 2021). However, in 2017, another study indicated that FTO preferentially demethylates N^6^,2′-O-dimethyladenosine (m^6^Am) rather than m^6^A (Mauer et al., 2017; Mauer and Jaffrey, 2018). Most recently, it was suggested that the substrate preference of FTO might depend on its cellular localization which varies between cell types. While m^6^A is the preferable target of FTO in the nucleus, cytosolic FTO demethylates particularly m^6^Am (Wei et al., 2018; Relier et al., 2021). Notably, this substrate selectivity may also be influenced by regulatory proteins such as ZBTB48 (zinc finger and BTB domain containing 48), a telomeric zinc finger protein that recruits FTO to specific m^6^A/m^6^Am-modified RNAs, thereby enhancing the specificity of FTO (Nabeel-Shah et al., 2024). Although FTO’s substrate preference appears context-dependent, the functional implications of these preferences across diseases are not fully understood. Further research is needed to clarify how FTO’s selective demethylation of m^6^A and m^6^Am influences pathophysiological processes, as distinct mechanisms may underlie FTO’s roles in metabolic, cardiovascular, and oncological disorders. Accurately distinguishing m^6^A- and m^6^Am-dependent pathways in specific disease contexts could provide valuable insights for targeted therapeutic strategies. However, due to the similarity of these modifications, some methods cannot differentiate between m^6^A and m^6^Am, complicating studies of FTO’s substrate-specific effects (Benak et al., 2023a). Besides m^6^A and m^6^Am, FTO has also an affinity to N^1^-methyladenosine (m^1^A) in transfer RNA (tRNA) (Mauer et al., 2017; Wei et al., 2018). Dynamic regulation of these epitranscriptomic modifications by FTO significantly affects the lifecycle of modified RNAs and consequently influences gene expression. Thus, epitranscriptomic regulations by FTO vastly affect cellular physiology and pathophysiology.

**FIGURE 1:**
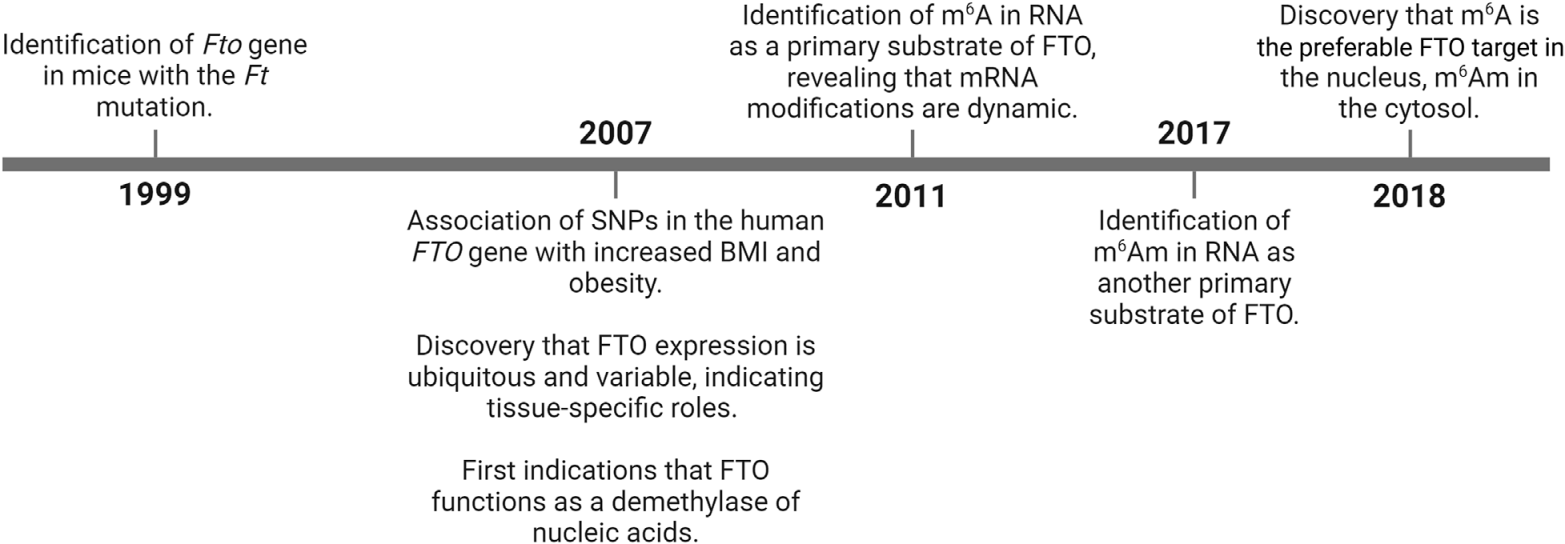
History of FTO research milestones. BMI – body mass index; FTO – fat mass and obesity-associated; m^6^A – N^6^-methyladenosine; m^6^Am – N^6^,2‘-O-dimethyladenosine; SNPs – single nucleotide polymorphisms.

The timeline of discoveries related to FTO is summarized in Figure 1.

## 2 Protein structure of FTO

The human *FTO* gene encodes a protein consisting of 505 amino acids with a molecular weight of 58,282 Da. Although the sequence of this protein is highly conserved across species, minor differences exist. For instance, the mouse *Fto* gene encodes a slightly shorter protein with 502 amino acids and a molecular weight of 58,007 Da. Similarly, the rat FTO protein also contains 502 amino acids, though its molecular weight is slightly lower at 57,972 Da (UniProt Consortium, 2023).

Structurally, FTO contains two main domains: the N-terminal domain (NTD) and the C-terminal domain (CTD) (Han et al., 2010). The NTD (residues 1–322) is catalytically active and includes binding sites for the metal cofactor, 2-oxoglutarate, and the methylated nucleobase. The CTD (residues 331–505 does not interact with FTO’s primary or secondary substrates but forms extensive contacts with the NTD (Khatiwada et al., 2022). The catalytic core of NTC is formed by a jelly-roll motif, characterized by a distorted double-stranded β-helix, which is supported on one side by two α-helices (α3 and α4) and on the other by a stabilizing loop between β5 and β6. The CTD’s structure includes a three-helix bundle with α7, α8, and α10, which extensively interacts with the NTD, providing essential structural stabilization (Han et al., 2010). This interaction between the NTD and CTD is necessary for FTO’s catalytic function, as the NTD alone is inactive. Disruption of the NTD-CTD interface leads to a loss of FTO’s catalytic capabilities, underscoring the essential role of inter-domain interactions in the enzyme’s function (Han et al., 2010; Khatiwada et al., 2022).

## 3 FTO in obesity

Obesity is characterized by an abnormal or excessive accumulation of fat, which can negatively impact health. High BMI is a major risk factor for many non-communicable diseases including diabetes, cardiovascular diseases (CVDs), and various cancers, which will be covered in the following chapters of this review. Unfortunately, the prevalence of overweight and obesity is reaching pandemic proportions, affecting 60%–70% of the adult population in industrialized countries and continuous to rise rapidly (Avgerinos et al., 2019; WHO. Obesity and overweight, 2021). Notably, FTO has been associated with obesity and each of the aforementioned diseases (Gholami, 2024).

The link between SNPs in the *FTO* gene and obesity has been uncovered through GWAS as mentioned in the previous chapter (Hinney et al., 2007; Frayling et al., 2007; Scuteri et al., 2007; Piwonska et al., 2022). Interestingly, most of these SNPs are in intronic regions, with introns 1 and 2 alone containing 89 identified variants. This observation has given rise to two hypotheses: either the introns within the *FTO* gene function as cis-regulatory sites affecting the expression of adjacent genes (such as *IRX3* or *RPGRIP1L*), or they serve as auto-regulators for the *FTO* gene itself (Azzam et al., 2022; Smemo et al., 2014; Stratigopoulos et al., 2014; Berulava and Horsthemke, 2010). Interestingly, research has shown that male individuals carrying *FTO* risk alleles respond differently to weight management interventions compared to females, with exercise leading to greater weight loss in *FTO* risk allele carriers compared to those who do not carry these alleles, but only in males (Wang W. et al., 2022).

Experimental studies confirmed the link between FTO and obesity also in animal models. Loss of FTO in mice resulted in reduced body weight and lower fat mass, while higher levels of FTO caused the opposite (Fischer et al., 2009; Church et al., 2009; McMurray et al., 2013; Church et al., 2010).

In individuals carrying *FTO* risk variants, the observed increase in BMI was primarily associated with higher energy consumption and diminished food satiety, rather than reduced energy expenditure (Speakman et al., 2008; Haupt et al., 2009; Wardle et al., 2008; Cecil et al., 2008; Wardle et al., 2009). However, altered energy expenditure has been also reported in mice with *Fto* deletions (Wu et al., 2021).

FTO is ubiquitously expressed, but the most prominent expression occurs in the brain, particularly within the hypothalamic nuclei responsible for the regulation of energy balance, such as the arcuate nucleus. The hypothalamic nuclei play a central role in regulating appetite and energy balance by integrating hormonal and nutrient signals that influence feeding behavior and metabolic processes. The arcuate nucleus is a key hypothalamic region that contains specialized neurons responsive to hormones like leptin and ghrelin, which signal states of satiety and hunger, respectively. These nuclei modulate energy intake and expenditure, acting as central coordinators of body weight and fat accumulation (Dhillo, 2007).

Expression of FTO in the arcuate nucleus is influenced by feeding and fasting cycles (Gerken et al., 2007). Selective alteration of FTO levels in the arcuate nucleus was able to influence food intake in rats (Tung et al., 2010). Inhibition of hypothalamic FTO activated STAT3 (signal transducer and activator of transcription 3) through ERK1/2 (extracellular signal-regulated kinase 1/2), which resulted in reductions in food intake and body weight (Hu et al., 2023). Moreover, it was reported that FTO colocalizes with the long isoform of leptin receptor within the arcuate nucleus and that leptin administration can result in a reduction of hypothalamic FTO levels both *in vitro* and *in vivo* (Wang et al., 2011). Several studies demonstrated that FTO promoted the hypothalamic leptin resistance induced by high-fat diet (Tung et al., 2015; Liu et al., 2024). Also, risk variants of the human *FTO* gene were associated with higher serum leptin levels (Mehrdad et al., 2020; Genis-Mendoza et al., 2020; Magno et al., 2018). Besides leptin, a link between FTO and hunger hormone ghrelin has been suggested as *Fto* knockout (KO) mice exhibited higher circulating ghrelin levels after a 16-h overnight fast. Moreover, FTO overexpression in cell cultures (MGN3-1 and HEK293T cells) reduced m^6^A methylation of ghrelin mRNA (*Ghrl*) and resulted in its upregulation (Karra et al., 2013). This association was also observed in humans carrying risk *FTO* alleles, whose peripheral blood cells exhibited an increased abundance of *FTO* and *GHRL* mRNA (Karra et al., 2013). However, in women with morbid obesity, the *FTO* risk variant was associated with decreased ghrelin levels in the postprandial period (Magno et al., 2018). This intricate interplay highlights the pivotal role of FTO in the neuroendocrine regulation of appetite and energy homeostasis.

Besides the regulation of food consumption and food satiety, FTO also plays a role in adipogenesis. FTO expression gradually decreased while m^6^A levels steadily increased during adipogenesis. Moreover, FTO regulated alternative splicing of adipogenic regulatory factor RUNX1T1 (runt-related transcription factor 1) modulating preadipocyte differentiation (Zhao et al., 2014). Another study showed that *Fto* KO in 3T3-L1 cells inhibited preadipocyte differentiation, while its overexpression enhanced the process (Zhang et al., 2015). Other experiments on 3T3-L1 cells presented that KO of *Fto* was linked with higher m^6^A levels on transcripts of early mitotic events regulators (*Ccna2*, *Cdk2*) and also autophagy-related transcripts (*Atg5*, *Atg7*), which led to their degradation, resulting in impairment of cell-cycle progression (Wu et al., 2018), autophagy (Wang X. et al., 2020), and inhibition of adipogenesis (Wu et al., 2018; Wang X. et al., 2020). Further research indicated that FTO is a target of NADP (nicotinamide adenine dinucleotide phosphate), which increases its activity, promoting m^6^A demethylation and adipogenesis (Wang L. et al., 2020). Merkestein et al. (Merkestein et al., 2015) reported that primary adipocytes and mouse embryonic fibroblasts (MEFs) derived from FTO-4 mice (*Fto* overexpression) exhibited increased potential for adipogenic differentiation, whereas MEFs derived from *Fto*-KO mice displayed reduced adipogenesis. Also in this study, the effect of FTO on adipogenesis appeared to be mediated via enhanced expression of the pro-adipogenic short isoform of RUNX1T1 (Merkestein et al., 2015). FTO deficiency has been also associated with browning, the conversion of white adipocytes into adipocytes with brown fat characteristics. It has been reported that FTO inhibited the expression of UCP-1 (uncoupling protein 1) in adipocytes, inhibiting the transformation of adipose tissue into brown adipose tissue (Tews et al., 2013). In line with this, a risk variant of the human *FTO* gene has been associated with a cell-autonomous shift from white adipocyte browning and thermogenesis to lipid storage, resulting in increased fat stores and body-weight gain (Claussnitzer et al., 2015). A recent study revealed that loss of FTO in adipose tissue can increase the m^6^A methylation of *Hif1a* (hypoxia-inducible factor 1 α) mRNA, which is recognized by m^6^A-binding protein YTHDC2, which promotes its translation resulting in increased protein abundance of HIF-1α. This well-known transcription factor then activates the transcription of thermogenic genes (*Ppaggc1a*, *Prdm16*, *Pparg*) and promotes *Ucp1* expression and the browning process (Wu et al., 2021).

Under physiological conditions, skeletal muscles serve as the main peripheral organs responsible for lipid utilization. Under pathological conditions, lipid accumulation becomes excessive. It has been suggested that AMPK (AMP-activated protein kinase) negatively regulates skeletal muscle lipid accumulation through FTO-dependent demethylation of m^6^A (Wu et al., 2017). Mechanistically, AMPK decreased FTO protein levels in C2C12 cells, therefore increasing m^6^A levels. A subsequent investigation revealed that m^6^A methylation upregulates skeletal muscle lipid accumulation, likely through upregulation of lipid synthase related genes and downregulation of lipolysis and oxidation related genes (Wu et al., 2017).

**FIGURE 2:**
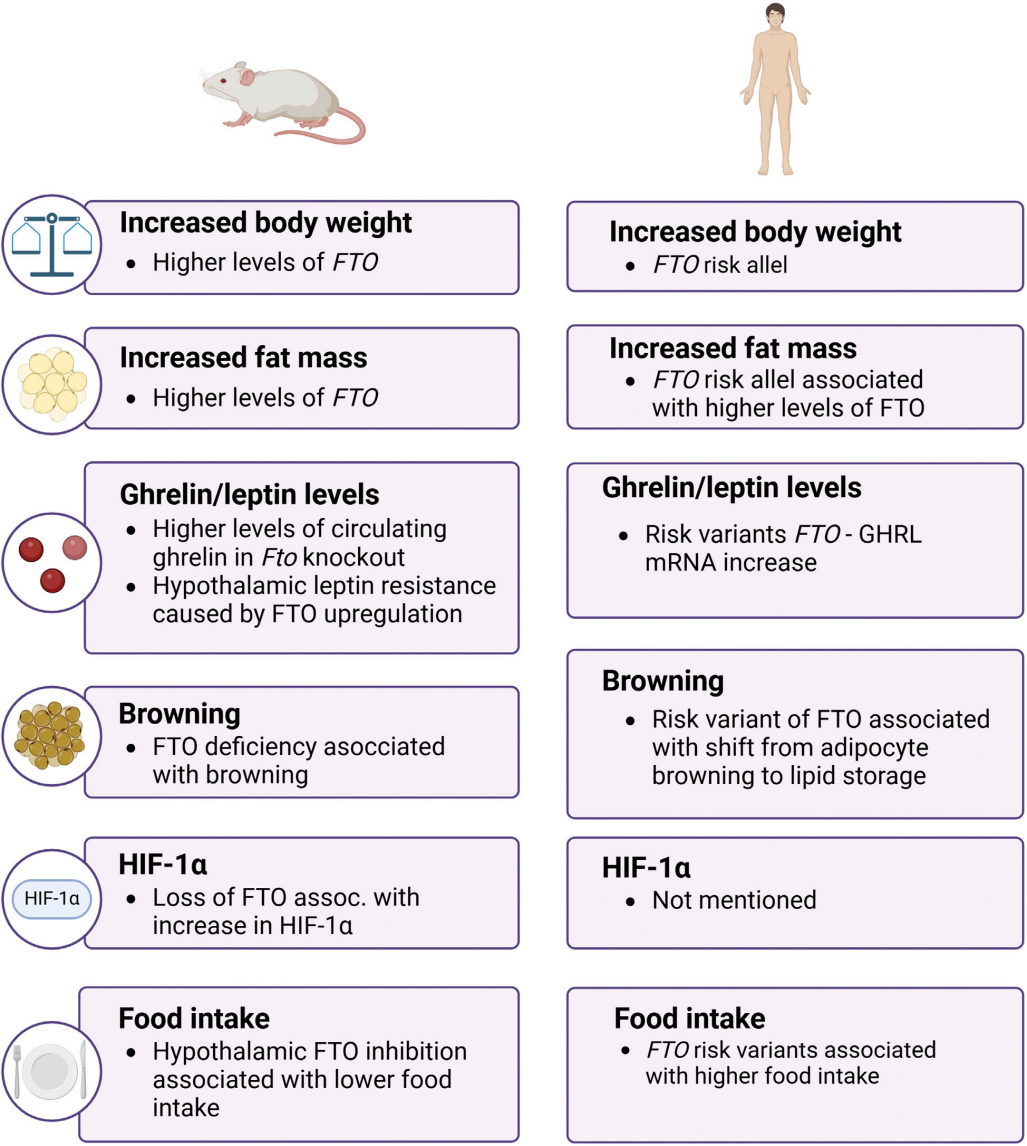
The role of FTO in obesity in mice and men. The image shows that FTO influences body weight, fat mass, and hormone levels in both mice and humans, with both species exhibiting increased obesity-related traits linked to FTO function. FTO – fat mass and obesity-associtated; GHRL – ghrelin; HIF-1α – hypoxia-inducible factor 1 α.

In conclusion, the *FTO* gene plays a pivotal role in the regulation of body weight and fat mass through complex mechanisms involving appetite regulation, energy balance, and adipogenesis (Figure 2). The intricate interplay between genetic variants of *FTO*, environmental factors, and metabolic pathways underscores the significance of this demethylase in the pathogenesis of obesity. Further research into the molecular mechanisms and potential therapeutic targets related to FTO could open new avenues for the prevention and treatment of obesity and its associated metabolic disorders.

## 4 FTO in diabetes

Diabetes mellitus is a widespread chronic disease with an increasing number of cases worldwide (WHO, 2022). It is well-established that obesity is a major risk factor for type 2 diabetes mellitus (T2DM), as excess adipose tissue promotes insulin resistance through increased secretion of inflammatory cytokines, free fatty acids, and other metabolic byproducts that disrupt insulin signaling. Consequently, the risk of T2DM rises linearly with increases in body mass index (Klein et al., 2022). T2DM exceeds type 1 diabetes mellitus (T1DM) and gestational diabetes mellitus (GDM) in frequency and accounts for approximately 90% of all diabetes diagnoses (Goyal and Jialal, 2023; Babakhanian et al., 2022). This heterogeneous systemic disease is mainly characterized by two factors: insufficient insulin secretion by pancreatic β-cells and insulin resistance of insulin-sensitive tissues (Galicia-Garcia et al., 2020). Pancreatic β-cells are specialized cells within the islets of Langerhans that play a central role in regulating blood glucose levels by producing and secreting insulin. In response to rising blood glucose levels, β-cells release insulin, a hormone essential for glucose uptake by tissues and overall glucose homeostasis. This process is finely tuned by various cellular mechanisms that detect glucose and other metabolic cues to ensure timely insulin secretion. Disruptions in β-cell function can lead to insufficient insulin production, a key factor in the development of diabetes mellitus (Ashcroft and Rorsman, 2012). The subsequent chronic hyperglycemia (a characteristic feature of T2DM) damages glucose-sensitive organs and leads to subsequent impairment of vital functions (Malone and Hansen, 2019).

The progression from obesity to diabetes is linked to FTO’s dual influence on energy balance and adipose tissue, where its regulatory effects can contribute to systemic metabolic dysfunctions, such as insulin resistance, that characterize diabetes. FTO’s influence on adipogenesis, for example, may intensify lipid storage and impair insulin sensitivity in peripheral tissues, laying the groundwork for β-cell dysfunction and chronic hyperglycemia.

According to numerous studies, carriers of several SNPs in the human *FTO* gene are genetically predisposed to T2DM, T1DM, GDM, and chronic diabetic complications (Benak et al., 2023b; Hubacek et al., 2018a; Younus et al., 2017; Hubacek et al., 2023; Ghafarian-Alipour et al., 2018; Chaudhary et al., 2024; Mosaad et al., 2024; Zhang et al., 2023; Amin et al., 2023; Amine Ikhanjal et al., 2023; Zano and Baig, 2022). However, this association is still controversial with significant interethnic differences (Younus et al., 2017; Sabarneh et al., 2018; Sarkar et al., 2021; Bakhashab et al., 2020; Bazzi et al., 2014; Nasser et al., 2019; Chauhan et al., 2011; Bressler et al., 2010; Sanghera et al., 2008; Vasan et al., 2014; Yajnik et al., 2009; Yu H. et al., 2023). Moreover, some variants were found to be protective against diabetes (Bressler et al., 2010).

Gene expression analysis in pancreatic islets collected from healthy individuals and T2DM patients discovered downregulation of *FTO* in the diabetic group (De Jesus et al., 2019; Taneera et al., 2018; Kirkpatrick et al., 2010; Taneera et al., 2024). In contrast to these results, *in vitro* experiments revealed that high glucose concentrations in Min6 cells (mouse β-cell line) increased gene expression of *Fto* (Bornaque et al., 2022). Another study showed that increased production of this demethylase in Min6 cells promoted reactive oxygen species (ROS) generation and led to NF-κB (nuclear factor kappa-light-chain-enhancer of activated B cells) activation, which resulted in the inhibition of insulin secretion (Fan et al., 2015). Impaired insulin release was observed in INS-1 cells (rat insulinoma cell line) after *Fto* silencing, however, ROS production rate was not affected in this case (Taneera et al., 2024). On the contrary, overexpression of *FTO* in human islets promoted insulin secretion and increased protein levels of key β-cell proteins: INS, PDX1, MAFA, and GLUT1 (Taneera et al., 2024) The observed discrepancies between specific animal cell lines and diverse human islets could be attributed to variations between species or the inherent diversity within islet cell populations.

Besides the role of FTO in the diabetic islets of Langerhans, FTO has been also regulated in many other diabetic tissues. In db/db mice, which are used as a model of T2DM and diabetic cardiomyopathy, downregulation of cardiac FTO on both gene and protein levels was observed, resulting in elevated m^6^A levels (Ju et al., 2021). FTO was described to promote the progression of diabetic nephropathy (Sun et al., 2022), and several SNPs in the *FTO* gene were associated with a significantly lower risk of nephropathy in T2DM patients (Montesanto et al., 2018). *FTO* polymorphism was also linked to a higher risk of diabetic retinopathy (Hsiao et al., 2021). FTO expression was elevated in diabetic mice retinas and systemic administration of FTO inhibitor FB23-2 exhibited therapeutic efficacy in mice with diabetic retinopathy (Chen et al., 2024).

Importantly, increased FTO levels were detected in the peripheral blood of patients with T2DM (Shen et al., 2015; Onalan et al., 2022). Furthermore, a connection between high FTO levels and the severity of T2DM has been suggested (Masoud Abd El Gayed et al., 2021). In white blood cells of T2DM patients, a positive correlation between high *FTO* gene expression and fasting glucose concentration was found (Yang et al., 2019). Collectively, these findings suggest that FTO levels in peripheral blood could potentially serve as a novel biomarker for T2DM in the future.

In conclusion, FTO plays a critical role in the pathogenesis of diabetes, influencing insulin secretion, insulin sensitivity, and the development of diabetic complications through diverse mechanisms. The association between genetic variants of *FTO* and diabetes underscores the complexity of the disease and highlights the potential of FTO as a biomarker and therapeutic target in managing diabetes and its complications.

## 5 FTO in cardiovascular diseases

CVDs are the leading cause of death worldwide, with obesity significantly elevating risk through mechanisms like adipose tissue accumulation in the myocardium, as well as promoting a pro-inflammatory and prothrombotic state (Ashraf and Baweja, 2013). Globally, CVDs contribute to approximately 18 million deaths each year (WHO. Cardiovascular diseases, 2021). Current knowledge suggests that FTO plays a comprehensive role in cardiovascular health, impacting processes from the initial development of heart tissue to heart disease progression, making FTO an intriguing molecular target for potential clinical interventions.

The growth, proliferation, and differentiation of cardiomyocytes are critically dependent on the precise regulation of gene expression, with epitranscriptomic modifications increasingly recognized as vital contributors to these processes (Liu et al., 2023; Han et al., 2021; Yang C. et al., 2021). Levels of FTO are regulated throughout the development of the heart in a sex-dependent manner (Semenovykh et al., 2022; Krejčí et al., 2023). Loss-of-function mutation in the human *FTO* gene was associated with a range of heart defects (ventricular septal defect, atrioventricular defect, patent ductus arteriosus), as well as hypertrophic cardiomyopathy (Boissel et al., 2009). Moreover, variants of the *FTO* gene were linked with various CVDs, including hypertension, myocardial infarction (MI), acute coronary syndrome, and increased risk of rejection in heart transplant patients (Ahmad et al., 2010; He et al., 2014; Liu et al., 2013; Doney et al., 2009; Hubacek et al., 2016; Hubacek et al., 2010; Hubacek et al., 2018b). Regulation of FTO levels was observed in MI and heart failure (HF) patients and corresponding animal models (Mathiyalagan et al., 2019; Shi et al., 2021; Zhang et al., 2021a; Zhang et al., 2021b; Hinger et al., 2021; Wen et al., 2022; Wang X. et al., 2022; Vausort et al., 2022; Liu et al., 2022). However, it's important to note the possibility that the regulation of FTO in the heart could vary with age: downregulation of FTO levels was observed in response to acute myocardial ischemia-reperfusion injury in elderly murine hearts but remained unaffected in young hearts (Su et al., 2021).

FTO activity has been associated with cardiac hypertrophy. Global *Fto* KO in mice resulted in cardiac hypertrophy development (Carnevali et al., 2014). However, *in vitro* studies showed that cardiomyocyte hypertrophy can be triggered also by a leptin-induced increase in FTO levels, while FTO knockdown with siRNA abolished this effect (Gan et al., 2013). Transverse aortic constriction (TAC), an experimental model of pressure overload-induced cardiac hypertrophy and HF, was associated with a reduction of cardiac FTO levels, while FTO overexpression was shown to attenuate the cardiac dysfunction following TAC (Zhang et al., 2021a; Li W. et al., 2022), particularly by regulating glucose uptake and glycolysis (Zhang et al., 2021a). Mice with a cardiomyocyte-specific KO of *Fto* exhibited an impaired cardiac function manifesting with a more severe reduction in ejection fraction and a higher degree of left ventricular dilatation compared to wild-type animals upon TAC (Berulava et al., 2020). A recent study showed that the beneficial effects of cinnamic acid treatment of hypertrophy and HF in TAC mice are at least partially mediated by increasing FTO expression (Cui et al., 2023). In contrast to these data, the negative role of FTO has been documented in mice with HFpEF (heart failure with preserved ejection fraction), where upregulated FTO levels were reduced by exercise training. Overexpression of this demethylase then canceled the benefits of exercise and promoted myocyte hypertrophy, apoptosis, and myocardial fibrosis (Liu et al., 2022). The association of FTO and myocardial fibrosis was also documented by other studies, albeit with opposite results. In mouse models of MI, overexpression of *Fto* resulted in the reduction of fibrosis (Mathiyalagan et al., 2019). A recent study demonstrated that antifibrotic effects of leonurine in rat cardiac fibroblasts are also mediated through upregulation of FTO, which in turn leads to a reduction in m^6^A methylation levels (Meng et al., 2024).

Loss of FTO has been linked with a proarrhythmic remodeling of the heart. Global deficiency of this demethylase in mice resulted in a phenotype characterized by higher heart rate and heart rate variability, susceptibility to stress-induced tachyarrhythmias, and altered ventricular repolarization (Carnevali et al., 2014). According to a recent study, decreased *FTO* gene expression was an important predictor of atrial fibrillation in patients with metabolic syndrome (Rafaqat et al., 2024). Suppression of FTO was also linked to myocardial inflammation and dysfunction in mice during endotoxemia (Dubey et al., 2022). Furthermore, recent research has highlighted the role of FTO in mitigating septic cardiomyopathy through the suppression of ferroptosis, thereby alleviating heart inflammation and dysfunction (Zeng et al., 2024)​. However, another study demonstrated that inhibition of FTO using the LuHui Derivative (LHD) compound alleviated the inflammatory response and injury in hyperlipidemia-induced cardiomyopathy in rats (Yu et al., 2021).

Mice on HFD exhibited decreased cardiac FTO levels, which were reversed by long-lasting intermittent fasting, a well-known cardioprotective intervention (Xu et al., 2022). Similarly, a recent study demonstrated that FTO levels were elevated in the left ventricles of rats after a 3-day fasting period. Subsequent *in vitro* experiments revealed that cardiomyocytes isolated from fasted animals exhibited reduced hypoxic tolerance after FTO inhibition (Benak et al., 2024). Several other publications have described the positive impact of FTO on the tolerance of cardiomyocytes to hypoxia (Deng et al., 2021; Shen et al., 2021; Ke et al., 2022; Hlavackova et al., 2018). A recent study demonstrated that FTO targets sarcoplasmic/endoplasmic reticulum Ca^2+^ ATPase 2a (SERCA2a), leading to preservation of calcium homeostasis for myocardial contractile function in MI (Yang H. et al., 2024). FTO has also exhibited cardioprotective effects against the cardiotoxic effects of different drugs, such as sunitinib or doxorubicin (Ma et al., 2022; Yang Y. et al., 2024; Yu P. et al., 2023).

These data show that FTO exerts both beneficial and detrimental effects on the heart, depending on the underlying conditions. Thus, more studies are needed to elucidate the complex effects of FTO on the biology of the cardiovascular system.

## 6 FTO in cancer

Obesity is associated with an increased risk for a range of malignancies, largely due to altered fatty acid metabolism, extracellular matrix remodeling, secretion of adipokines and various hormones, immune dysregulation, and chronic inflammation. Although these mechanisms contribute to cancer development and recurrence, the exact relationship between obesity and cancer risk remains incompletely understood (Pati et al., 2023). According to the International Agency for Research on Cancer Working Group, there is convincing evidence that high body weight is linked to a higher risk for cancer of at least 13 anatomic sites, including endometrial, esophageal, renal and pancreatic adenocarcinomas, gastric cardia cancer, meningioma, multiple myeloma, colorectal, postmenopausal breast, ovarian, gallbladder, and thyroid cancers (Avgerinos et al., 2019; Lauby-Secretan et al., 2016). Given that FTO plays a crucial role in metabolism and obesity, it is not surprising that FTO dysregulation also significantly impacts tumorigenesis. Across a broad spectrum of cancer types, FTO is commonly found to be upregulated, serving as a crucial promoter of tumor progression (Li Y. et al., 2022). For instance, FTO has been reported as an oncogene in metastatic endometrial carcinoma, gastric cancer, bladder cancer, hepatocellular carcinoma, or acute myeloid leukemia; however, it also acts as an anti-oncogene in gastric cancer (An and Duan, 2022; Li et al., 2017).

Research into the relationship between FTO and cancer risk began shortly after SNPs in the human *FTO* gene were linked to obesity. However, establishing a straightforward link between polymorphic variants of *FTO* and cancer has proven difficult because many variables, such as ethnicity or the genetic origin of the tumors being compared (e.g., spontaneous vs hereditary tumors) (Azzam et al., 2022; Hernández-Caballero and Sierra-Ramírez, 2015). Despite this, several *FTO* SNPs were linked to higher or lower risk of cancers. For instance, already in 2009, Brennan et al. (Brennan et al., 2009) linked a variant of *FTO* gene to a lower risk of lung cancer and a slightly increased risk for kidney cancer. Later research associated *FTO* polymorphisms in some races with higher risks of pancreatic cancer (Tang et al., 2011; Pierce et al., 2011), endometrial and breast cancer (Delahanty et al., 2011; Lurie et al., 2011; Kaklamani et al., 2011), melanoma (Iles et al., 2013), or colorectal cancer (Gholamalizadeh et al., 2023).

The important hallmark of cancer is uncontrolled cell proliferation due to changes in metabolism and signaling pathways. These metabolic changes provide the energy and materials needed for cell growth and adaptation to tumor microenvironment. Recent studies showed that m^6^A modification is widely involved in the metabolic recombination of tumor cells (An and Duan, 2022). The mTOR (mammalian target of rapamycin) signaling pathway is a key regulator of tumor metabolism (Mirabilii et al., 2020). Several studies have already connected FTO to mTOR signaling (Li et al., 2018; Wang et al., 2017). For instance, stabilization of miR-139-5p by FTO supressed prostate cancer cell malignancies by inactivating the PI3/Akt/mTOR signal pathway (Azhati et al., 2023). Another recent study reported that FTO-induced upregulation of flotillin-2 contributed to cancer aggressivness in diffuse large B-cell lymphoma by activating PI3K/Akt/mTOR pathway (Zhang et al., 2024). Thus, the role of FTO in regulating the mTOR pathway is ambiguous, as it can lead to both activation and inactivation, resulting in negative and positive outcomes. FTO demethylates also the most important transcription factor and extensive nuclear oncogene MYC (MYC proto-oncogene, also named c-Myc). It has been reported that FTO can reduce the methylation of MYC in gastric cancer cells and stabilize its expression, ultimately resulting in augmented proliferation, migration and invasion of gastric cancer cells *in vitro* (Yang Z. et al., 2021). However, another study showed that MYC activated in Epstein-Barr virus-associated gastric cancer elevated FTO expression by binding to the FTO promoter. The increase in FTO levels was then associated with depressed cell metastasis, aggressiveness, and overall better clinical outcomes (Xu et al., 2023). Evidently, FTO’s role in gastric cancer can be both positive and negative, depending on the specific molecular and pathological context. This duality underscores the importance of context in understanding the function of molecular players in cancer biology.

Additionally, FTO has been implicated in resistance to chemo-radiotherapy. In cervical squamous cell carcinoma, FTO upregulates β-catenin by reducing m^6^A modification, leading to enhanced resistance to chemo-radiotherapy (Zhou et al., 2018). Similarly, in neuroblastoma, FTO has been shown to influence sensitivity to chemotherapeutic drugs, enhancing the response to paclitaxel while reducing sensitivity to etoposide, indicating a drug-specific role in chemotherapy resistance (Lin et al., 2024). This highlights the critical role of the FTO in modulating not just tumor growth but also drug resistance, making it a potential target for improving cancer therapy outcomes.

In conclusion, the role of FTO in cancer is multifaceted, influencing not only tumor growth and progression through metabolic reprogramming and m^6^A RNA modification but also impacting treatment outcomes by contributing to resistance mechanisms such as chemo-radiotherapy. Its involvement in key pathways like PI3K/Akt/mTOR and its regulation of oncogenes such as MYC and β-catenin further emphasize the importance of context when evaluating FTO’s function in various cancer types. As research continues to uncover the complexities of FTO’s role, it may offer novel therapeutic opportunities to target cancer growth and overcome treatment resistance.

## 7 FTO as a pharmacological target

Various inhibitors targeting FTO demethylase with a potential to increase m^6^A and m^6^Am methylation are currently available.

Rhein, an anthraquinone compound derived from the rhubarb plant (*Rheum palmatum*), was recognized as the first cell-active FTO inhibitor in 2012 (Chen et al., 2012). However, rhein is not a specific inhibitor as it acts also on other molecular targets (Henamayee et al., 2020), e.g., histone deacetylases (Barbosa et al., 2020). This lack of specificity exemplifies a central challenge in FTO inhibitor development: achieving selectivity to avoid unwanted effects on other enzymes and pathways.

Meclofenamic acid (MA), a well-recognized drug for its anti-inflammatory properties via cyclooxygenase inhibition, has been also described to inhibit the FTO enzyme (Huang et al., 2015). Entacapone, a reversible catechol-*O*-methyltransferase inhibitor used in the treatment of Parkinson’s disease, has been shown to bind FTO and inhibit its activity as well (Peng et al., 2019). While these compounds have shown efficacy in inhibiting FTO, their primary pharmacological targets lie elsewhere, highlighting the difficulty of repurposing drugs with established indications.

Besides these non-specific drugs, the compound MO-I-500 was introduced as a specific inhibitor of FTO (Zheng et al., 2014). Fluorescein and its derivatives were presented as bifunctional molecules for either inhibiting or labeling FTO (Wang et al., 2015). Additionally, selective inhibition of FTO was also achieved with other small molecule inhibitors, including FB23, FB23-2, CS1, CS2, Dac51, LHD, FTO-02, FTO-04, N-phenyl-1H-indol-2-amine, MU06, radicicol, CHTB, N-CDPCB, nafamostat mesilate, NOG, PDCA, or saikosaponin D (Yu et al., 2021; Toh et al., 2015; He et al., 2015; Svensen and Jaffrey, 2016; Huang et al., 2019; Su et al., 2020; Huff et al., 2021; Qin et al., 2022; Liu et al., 2021; Padariya and Kalathiya, 2016; Wang et al., 2018; Qiao et al., 2016; Han et al., 2019; Aik et al., 2013; Sun et al., 2021; Qiu et al., 2023). Each of these molecules showcases different approaches to enhancing selectivity through structural modifications, though issues such as off-target binding and stability have limited their progression beyond experimental stages. Developing compounds that specifically target FTO in relevant disease tissues without systemic side effects remains a critical goal for therapeutic applications.

Recent advances in structural biology have allowed for the design of more selective FTO inhibitors based on its catalytic domain, an approach that has improved specificity and facilitated the development of promising drug candidates. Challenges like ensuring high specificity without off-target effects, achieving effective tissue targeting, and managing toxicity–as well as optimizing bioavailability, half-life, and tissue localization–remain critical hurdles for the clinical translation of these compounds. Consequently, FTO inhibitors are mainly used in experimental settings and have not advanced to clinical application, primarily due to these specificity, pharmacokinetic, and localization limitations (Huff et al., 2021). Notably, STC-15, an inhibitor of the m^6^A writer METTL3, has recently become the first RNA-modifying enzyme inhibitor to enter clinical trials (NCT05584111) (Medicine, 2024). This milestone underscores the growing potential of RNA-modifying enzyme inhibitors, suggesting that the clinical advancement of FTO inhibitors may soon follow.

In summary, while the development of FTO inhibitors holds promise, the future identification of clinically effective and highly selective, tissue-targeted FTO-targeting agents is critical. Success in this area will be essential for creating novel treatments for various diseases, requiring close integration of structural biology insights, tissue-specific targeting strategies, and careful optimization to ensure drug-like properties amenable to clinical use.

## 8 Conclusion and perspectives

This review thoroughly discusses the critical role of the FTO protein in both health and disease, with a particular focus on its involvement in obesity and related non-communicable diseases such as diabetes, cardiovascular disease, and cancer. FTO, a key enzyme in the regulation of RNA modifications, influences a variety of physiological processes including RNA splicing, export, decay, and translation. Its dysregulation is linked to obesity through mechanisms affecting energy balance, appetite, and adipogenesis.

The relationship between FTO and various diseases is complex. In obesity, FTO is associated with increased food intake and reduced satiety, contributing to higher BMI. It also plays a significant role in adipogenesis and the regulation of thermogenesis, further emphasizing its importance in metabolic disorders.

In diabetes, FTO influences insulin secretion and sensitivity, with genetic variants linked to an increased risk of T2DM. Additionally, FTO’s role in diabetic complications, such as nephropathy and retinopathy, suggests it could serve as a potential biomarker or therapeutic target.

FTO’s involvement in CVDs is multifaceted, with evidence suggesting both protective and detrimental effects depending on the context. It is implicated in cardiac hypertrophy, heart failure, and myocardial ischemia-reperfusion injury, among other conditions.

Finally, FTO’s role in cancer is discussed, particularly its impact on tumor metabolism and proliferation. While FTO is generally upregulated in various cancers, contributing to tumor progression, its effects can vary depending on the type and context of the cancer. The role of FTO in CVDs and cancer exemplifies its broad influence on shared mechanisms, including cell proliferation and metabolic regulation, highlighting its specificity in disease manifestation while underscoring its universal function across diverse cell types, from cardiovascular to tumor cells.

**FIGURE 3:**
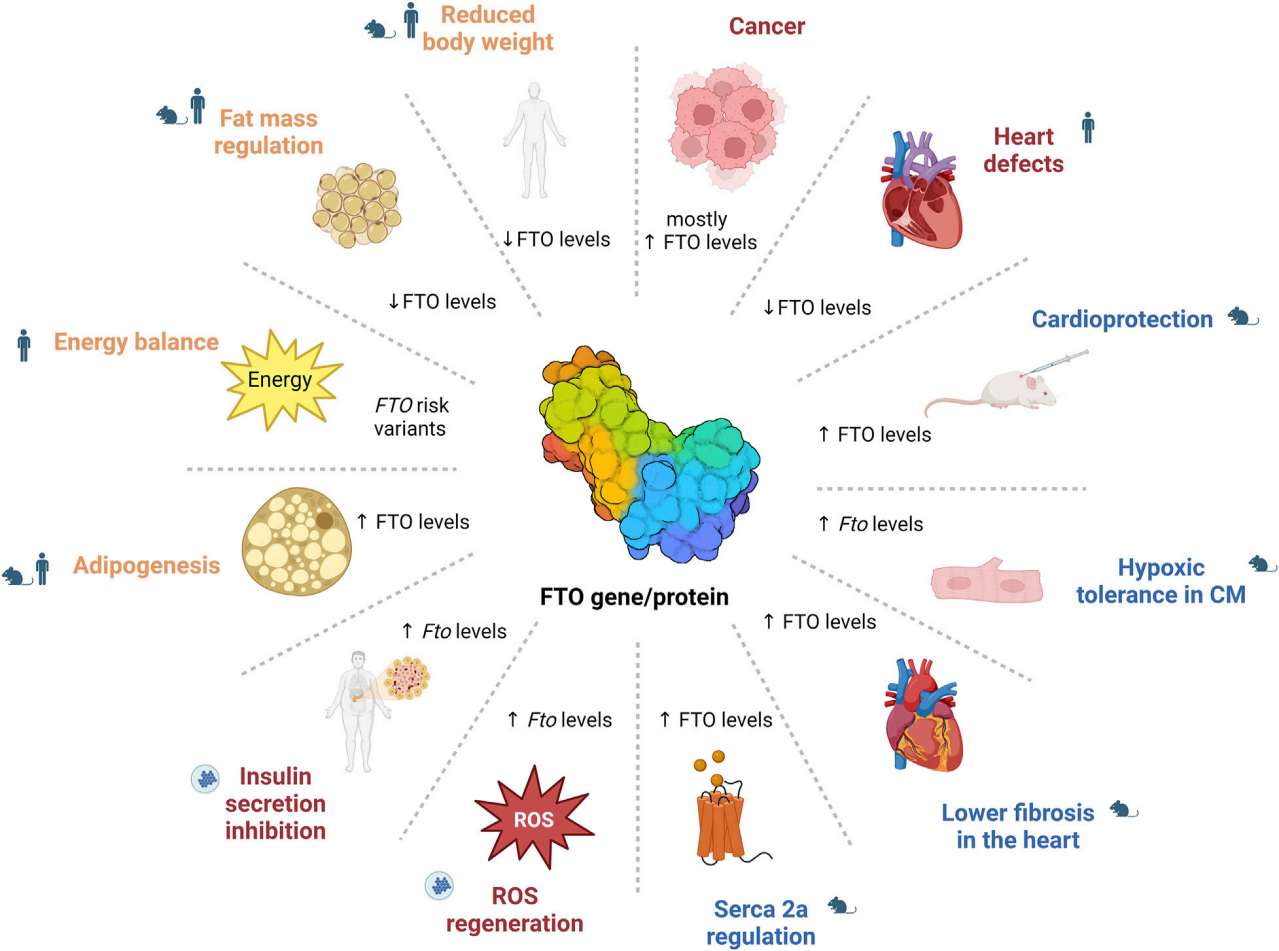
The role of FTO in health and disease. The image illustrates the diverse roles of FTO, showing that altered FTO levels affect various physiological and pathological processes, including energy balance and adipogenesis (e.g., through STAT3/ERK1/2 pathway or AMPK pathway regulation), diabetes (e.g., NF-κB pathway regulation), cardiovascular functions (e.g., SERCA2a regulation) and cancer (e.g., PI3K/Akt/mTOR pathway or MYC oncogene regulation), demonstrating its broad impact across the metabolic functions. CM – cardiomyocytes; FTO – fat mass and obesity-associated; ROS – reactive oxygen species.

Overall, this review underscores the importance of FTO as a central player in the epitranscriptomic regulation of health and disease (Figure 3). However, several challenges remain before these research findings can be implemented in clinical settings. A major obstacle in biomedical science is translating discoveries from laboratory animals, such as mice and rats, to human physiology. Substantial metabolic differences between rodents and humans–such as a 6.4-fold higher metabolic rate and a 9.6-fold faster protein turnover in rats (Agoston, 2017) – suggest species-specific variations in FTO function, given its role as a key metabolic regulator. These interspecies differences in FTO biology remain insufficiently understood, highlighting the need for further investigation to ensure the relevance of laboratory findings for clinical applications. Given FTO’s broad impact on physiology and pathophysiology, it remains a promising target for therapeutic interventions across a range of conditions. However, additional research is essential to fully elucidate its complex mechanisms and to develop effective, selective inhibitors that are suitable for clinical use. It is crucial that such therapeutic strategies are designed with precision to ensure that targeting FTO in one context does not disrupt its regulatory functions in other tissues or processes, thus avoiding potential adverse effects, such as treating CVDs at the risk of promoting cancer or other pathologies.
